# Towards a passive limitation of particle surface contamination in the Columbus module (ISS) during the MATISS experiment of the Proxima Mission

**DOI:** 10.1038/s41526-020-00120-w

**Published:** 2020-10-20

**Authors:** Laurence Lemelle, Lucie Campagnolo, Eléonore Mottin, Denis Le Tourneau, Emmanuel Garre, Pierre Marcoux, Cécile Thévenot, Alain Maillet, Sébastien Barde, Jérémie Teisseire, Guillaume Nonglaton, Christophe Place

**Affiliations:** 1 Univ Lyon, ENS de Lyon, Univ Claude Bernard, CNRS, Laboratoire de Géologie de Lyon-Terre Planètes et Environnement, Lyon, France; 2 MEDES-IMPS for CADMOS, Toulouse, France; 3 Univ Lyon, ENS de Lyon, CNRS, Laboratoire de Physique, Lyon, France; 4 Surface du Verre et Interface, UMR CNRS/Saint-Gobain, Aubervilliers, France; 5 Université Grenoble Alpes, CEA, LETI, DTBS, Grenoble, France; 6 CNES, Toulouse, France

**Keywords:** Biotechnology, Biophysics

## Abstract

Future long-duration human spaceflight calls for developments to limit biocontamination of the surface habitats. The MATISS experiment tests surface treatments in the ISS’s atmosphere. Four sample holders were mounted with glass lamella with hydrophobic coatings, and exposed in the Columbus module for ~6 months. About 7800 particles were detected by tile scanning optical microscopy (×3 and ×30 magnification) indicating a relatively clean environment (a few particles per mm^2^), but leading to a significant coverage-rate (>2% in 20 years). Varied shapes were displayed in the coarse (50–1500 µm^2^) and fine (0.5–50 µm^2^) area fractions, consistent with scale dices (tissue or skin) and microbial cells, respectively. The 200–900 µm^2^ fraction of the coarse particles was systematically higher on FDTS and SiOCH than on Parylene, while the opposite was observed for the <10 µm^2^ fraction of the fine particles. This trend suggests two biocontamination sources and a surface deposition impacted by hydrophobic coatings.

## Introduction

International space agencies plan to advance human spaceflight through a continued presence in low-Earth orbit (LEO), human missions to cis-lunar space and the lunar surface, and missions to Mars^1,2^. In this context, the hazardous risks incurred by the astronauts and the equipment’s integrity are challenging in several respects^3–5^. Microorganisms can develop over long durations some resistance, unknown mutations to the used disinfectants and antibiotics, and virulence^6–8^, while the cabin’s bio-contamination by the irreducible microflora of the crew is unavoidable^5,9,10^. Microorganisms that do not represent severe health hazards for healthy people may become a risk for astronauts due to the dysregulation of their immune function^11^. On board, air and water are transmission routes of pathogens that are controlled by filtration systems and monitored both on ground and in-flight by microbial analyses. Surfaces also constitute a source of microorganisms which abundances and diversity are highly variable and controlled^12–15^. Currently, this particular risk is mitigated by a cleaning strategy that consists of manually wiping the surface with disinfectants. Besides being time-consuming and laborious, it is inefficient for surfaces in inaccessible spaces^3^. This risk is increased in spacecraft by longer isolation and a greater reliance on an increasing number of closed-loop life support systems^12,13,16,17^. Furthermore, future long-duration exploration scenarii will include dormancy periods, where the spacecraft is left unmanned and not sterilized, thus requiring the development of autonomous microbial monitoring and control systems^18,19^. It has been discovered that an efficient approach to reduce the risks associated with microorganisms is to intervene in the design phase of new spacecraft^20^. Choosing materials with surfaces that do not contribute to microbial growth and spread is already a prerequisite^1^. Developing sustainable materials and equipment that reduce microbial growth and spread on surfaces is a natural next step for new spacecraft generation for longer duration exploration^21^.

Environmental solid surfaces increase the survival ability and infectiosity of microorganisms by their role as sources of nutrients and holders that support the development of abundant and complex communities. Once in a biofilm, microorganisms are protected from inhospitable environmental variations and from killing by antibiotics and disinfectants. In the human body, they are at the root of persistent and chronic bacterial infections^22^. Biocontaminated surfaces have been assessed to be infection foci and transmission routes of pathogens by indirect contact in healthcare settings^23–26^. Biofilm growth in spacecraft under microgravity has been observed experimentally^27^ and established to be favorable compared with ground controls, with notable increases of the number of viable cells, biomass, and thickness^28^. Metabolic fungal activities on MIR and in the early days of ISS were also identified to be at the origin of equipment degradation by corrosion^29–31^.

Several strategies can be conceived to limit surface biocontaminations. Bactericidal surfaces have the advantage of killing bacteria, but the accumulation of dead bacterial components or extracellular polysaccharides on the surface may paradoxically fuel bio-contamination in the long term. Furthermore, evaluating how the mechanism of disruption of the metabolic processes of the microorganism may work on human cells is a considerably expensive pre-requisite. In this respect, surfaces that reduce microbial attachment and repel microorganisms would avoid this drawback and additionally avoid microorganisms becoming trapped in the air filtration system. Such surfaces need to be designed using few compounds firmly anchored to the surface, and having as low chemical and nanoparticle toxicities as possible. As microorganisms are most likely transported under microgravity in droplets of hydrous solutions and because self-cleaning may be further implemented, hydrophobic coatings are considered an effective first-line^32^. The strategy here is not to reduce the strength of adhesion on a surface of a microorganism suspended in a fluid, which has been documented to involve multiple electrostatic and hydrophobic forces^33,34^, which vary over time. By reducing the contact area of water droplets and floating condensates possibly biocontaminated^14^ on surfaces, the hydrophobicity allows for the repulsion of water from the surface and thereby limits the surface contamination, prior to any species-dependent interaction of a microorganism with a surface.

The Matiss (Microbial Aerosol Tethering on Innovative Surfaces in the International Space Station) experiment was designed to investigate if hydrophobic coatings already implemented in numerous industrial fields could be applied to spacecrafts to limit bio-contamination. During the Matiss experiment, surfaces were exposed over long periods of time on the International Space Station using a holder designed for this application. Once returned, the exposed surfaces were analyzed by optical microscopy in the returned, and confined holder and all the particles present on the surfaces were listed. This approach impedes gathering information about the abundances and speciation of the microorganisms present on the surfaces using staining or swabbing-based techniques. However, by setting sights on the particulate contaminations, this approach provides the possibility of tracing further the different sources and routes of the surface biocontamination in the ISS. In this study, we report on the diversity of the particles observed on surfaces exposed for 6 months in the Columbus module.

## Results

### Exposure of surfaces to the Columbus atmosphere

We report on the diversity of particles collected at two sites with a low frequency of astronaut contact and good airflow in the Columbus module, on four types of surface coating that were exposed for ~6 months. The fluid dynamics in the environment of the two sites is documented (see “Materials and methods”, “Sampling with the MATISS sample holder”)^20^. The air exchange was performed with eight inlets and one large return grid, with a flow rate value of ca. 400 m^3^/h, about one order of magnitude higher than in ground ventilation, with velocity values of the laminar airflow in the range of 10–40 ft/min near the holders.

**Fig. 1:**
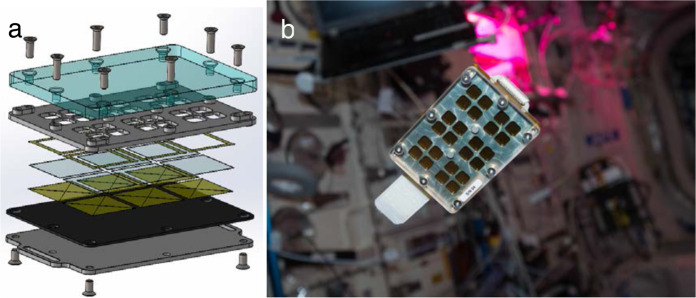
The MATISS sample holder. a Exploded-view drawing of the lamella holder showing the following top-down series: the polycarbonate lid (in light blue), the aluminum grid (in grey) ensuring the air circulation in a 2 mm-thick interspace on the side of the lid and the encasing and exposure of the lamella to the air on the other side. Kapton seals were fixed on both sides of the lamella to avoid any direct contact with the lamella. A Vitton plate was then adjoined to the aluminum mounting base with two slots to plug Velcro bands. **b** Photograph of the sample holder (8.5 cm × 6cm × 1.2 cm) before installation on the *Return Grid* Sensor *Housing* in the port-side cone of the *Columbus* module of the ISS. Photograph courtesy of NASA/ESA permissible to use within the public domain.

These surfaces were hydrophobic coated glass surfaces (FDTS, SiOCH, and Parylene) and a hydrophilic surface of the non-treated clean glass (see “Materials and methods”). The airborne particles that contaminated the exposed surfaces were collected using the MATISS sample holder (Fig. 1). This sample holder was designed with three aims: high operability and limited need of crew time, a safe long-term and unattended exposure of glass surfaces to the ISS atmosphere, and the possibility of running optical imaging of the particles confined within the sample holder (See “Data Availability” section).

In practice, this sample holder can be considered as a vented container with a slit between a transparent lid and the glass surfaces, allowing a laminar airflow on the surface of the mounted glass lamellae. The transition from the “laboratory-confined” state to the “ISS-exposed” state of the glass surfaces was manually operated by removing a Kapton tape that sealed the slit, and reversely by repositioning the Kapton tape.

### The diversity of the surface particles and the potential foci of infection

The size and number of particles collected within the MATISS sample holder after six months of exposure in the Columbus module were observed across the confined device by tile scanning optical microscopy at two different magnifications (Materials and methods).

At low magnification, particles with an area value bigger than 50 µm^2^ were identified.

**Fig. 2:**
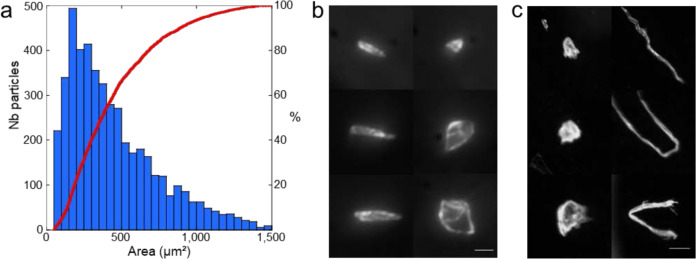
Surface contamination by coarse particles. **a** Particle size distribution histogram (blue bars) with the area in μm^2^ and cumulative particle size curve (red) for 4678 particles. **b**, **c** Mosaic of optical images recorded at high magnification displaying typical shapes of coarse (left, scale bar is 10 µm) and macroscopic (right, scale bar is 100 µm) particles (area > 1500 µm^2^).

The particle size distribution and the corresponding cumulative curve (Fig. 2a) of 12 lamellae display a monomodal distribution of 4678 particles with area values in the range of 50–1500 µm^2^ with the most probable size equal to ~155 µm^2^. This corresponds to an average density of fewer than two particles per square millimeter (1.6 ± 0.2 particle mm^−2^, Supplementary Fig. 1).

The sharp focus all over the particle area points out their flatness (depth of field » 55 µm in Fig. 2b, Supplementary Fig. 2) whatever their elongation ratio. The very flat shape of the particles (thickness < 1 µm) with straight linear sides forming a polygonal shape and an area bigger than 15 µm × 15 µm are consistent with the desquamated scales of a single or a few keratinized corneocytes from the astronauts’ epidermises^35,36^. The origin of the most abundant smaller particles is difficult to ascertain based on shape criteria only and they may have been inherited from an inorganic mineral source or have been produced by the partial degradation of the biggest particles. Above the 1500 µm^2^ threshold (Fig. 2c), fewer than 180 sub-millimeter particles were observed, among which one distinct morphotype could be unambiguously differentiated based on the elongation ratio. On the longitudinal view of the fibers, they appear to have a ribbon structure irregularly twisted, with a non-circular and irregular diameter of <30 µm and variable lengths. They could seemingly be textile fibers, either cellulosic from clothes, or polymeric and fiberglass from the Beta-cloth and the multi-layers insulation (MLI) (Supplementary Fig. 3). The largest round and thick particles display complex 3D structures and are therefore difficult to attribute to a specific source of contamination.

**Fig. 3:**
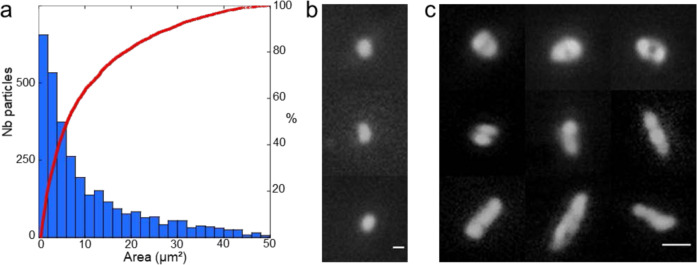
Surface contamination by fine particles. **a** Particle size distribution histogram (blue bars) with the area in μm^2^ and cumulative particle size curve (red) for 3175 particles **b**, **c** Mosaic of optical images displaying single particles with an area smaller than 5 µm² (left, scale bar is 2 µm) and segmented round and elongated particles (right, scale bar is 5 µm).

At high magnification, particles with an area value as small as 0.50 µm^2^ were observed. The particle size distribution and the corresponding cumulative curve for the 4 lamellae (Fig. 3a) displayed 3175 particles with area values in the range of 0.50–50 µm^2^. This corresponds to an average density of about 3.3 particles per square millimeter.

About half of the population has an average area value and a shape consistent with those of a single *cocci* (Fig. 3b) while the others, being either round or elongated (Fig. 3c, Supplementary Fig. 4), often display constriction figures, consistent with division features of filamentous microbial cells or *cocci*.

We therefore observed two types of contamination on the exposed surfaces inside the ISS: (i) macroscopic particles that can hold microbial cells on their own surface and which number can probably be restricted by sweep operation and (ii) microscopic particles among which several could be remnants of microbial cells deposited and strongly adsorbed onto the surfaces.

### The diversity of surface particles on the different hydrophobic coatings

**Fig. 4:**
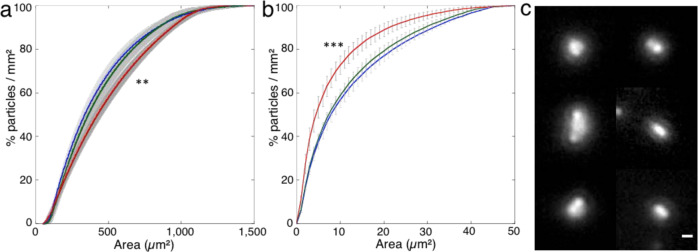
Surface contamination and surface treatments. **a** Cumulative particle size function of coarse particles (50 µm² < Area < 1500 µm^2^) in percentage per Area unit for FDTS (blue), SiOCH (green) and Parylene (red) surface coatings. Counts of particles measured from images recorded at low magnification. Statistical differences between parylen and either FDTS or SiOCH distributions were examined by unpaired Student’s *t*-test and found to be significantly different (**p-values < 0.01), contrary to that of FDTS and SiOCH (*p* = 0.4). **b** Cumulative particle size function of fine particles (0.5 µm² < Area < 50 µm²) in percentage per Area unit for FDTS (blue), SiOCH (green) and Parylene (red) surface coatings. Counts of particles measured from images recorded at high magnification. Error bars represent the standard deviations of the data (see description in the text). Statistical differences between parylen and either FDTS or SiOCH distributions were examined by unpaired Student’s *t*-test and found to be significantly different (***p-values < 0.001) contrary to that of FDTS and SiOCH (*p* = 0.13). **c** Mosaic of optical images displaying a bright halo around single particles (scale bar is 2 µm).

The diversity of the surface particles formed on the different coatings deposited on the glass lamella distributions was investigated. The three coatings FDTS, SiOCH, and Parylene, of which hydrophobicity is related to the water contact angle decreasing from 110° to 87° (see section Surface coatings in “Materials and methods”), were analyzed. First, the relative fractions of the particles compared with the area of the particles were compiled for each type of coating and displayed as a cumulative particle size function (Fig. 4). An average function from three of the exposed sample holders and the corresponding standard deviation for each of the area fractions, used here as error bars, were evaluated. It was also preliminarily verified that the coarse particle density values measured in the three sample holders for each type of coating were quite comparable (standard error lower than 15%, (see Table 1)). The main results are reported for the coarse (Fig. 4a) and the fine (Fig. 4b) particles, respectively.

**Table 1:** Coarse particle density values, *d* (particles/mm^2^), measured in two sample holders exposed near the Return Grid Sensor Housing (RGHS) and one sample holder on the European Physiology Modules Facility (EPM) front panel, on three different coatings (FDTS, SiOCH, Parylene).

	RGHS	RGHS	EPM	<*d*>
FDTS	1.99 ± 0.55	3.25 ± 0.95	2.12 ± 0.43	2.45 ± 0.64
SiOCH	1.54 ± 0.65	1.39 ± 0.51	1.22 ± 0.24	1.38 ± 0.47
Parylene	1.37 ± 0.10	1.70 ± 0.33	0.90 ± 0.37	1.33 ± 0.27
<*d*>	1.63 ± 0.32	2.11 ± 0.99	1.41 ± 0.63	

As regards the coarse particles (50 µm^2^ < Area < 900 µm^2^) (Table 1), the fractions of particles with area values lower than 200 µm^2^ are not significantly different (i.e., within less than one standard deviation). The fractions on the glass lamellae coated with FDTS and SiOCH are systematically higher than the fractions observed on the glass lamellae coated with Parylene for the area values in the 200–900 µm^2^ range. The difference can be ascribed to a higher fraction of the smallest particles on FDTS and SIOCH. The average particle densities are almost double on the FDTS than on SiOCH and Parylene.

As regards the fine particles (0.5 µm^2^ < Area < 50 µm^2^) (Table 2), the fractions of particles are not significantly different on the glass lamellae coated with FDTS or SiOCH (i.e. within less than one standard deviation) (Fig. 4b). These fractions are systematically lower than the one observed with Parylene, on which the fractions of the low-area particles (Area < 10 µm^2^) is higher. The higher hydrophobicity of FDTS and SIOCH disfavors contamination by small particles probably brought to the surface through water droplet deposition. Some droplets transportation is indeed supported by the regular circular halo observed around a few particles that could have been formed by the drying of a droplet on the surface (Fig. 4c).

**Table 2:** Fine particle density values, d (particles/mm²), measured in two sample holders exposed near the Return Grid Sensor Housing (RGHS) and one sample holder on the European Physiology modules Facility (EPM) front panel, on three different coatings (FDTS, SiOCH, Parylene).

	RGHS	RGHS	EPM	<*d*>
FDTS	1.43	8.35	4 ± 1.91	4.45 ± 3.07
SiOCH	2.18	1.07	5.98 ± 1.94	3.80 ± 2.79
Parylene	3.45	5.98	2.02 ± 0.49	3.37 ± 1.89
<*d*>	2.36 ± 1.02	5.13 ± 3.72	4 ± 1.98	

## Discussion

This study establishes experimental proof-of-concept that the MATISS sample holder is useful and adequate for investigating the particulate contamination after long-term exposure of surfaces in the ISS habitat once returned to ground. The low density of only a few particles per mm^2^ observed after six months’ exposure in one of the dirtiest locations in an instrumental module indicates relatively clean surfaces, corresponding to a Surface Cleanliness by Particle concentration of class 6 (>1 µm)^37^. However, if this rate is extrapolated to the foreseen lifetime of a spacecraft’s cabin of several decades, the final coverage of a surface reaches a value higher than 2% (2.2%) in 20 years, which is well above the reported safety threshold for electronic equipment^9,12,35^.

The diversity of the contaminating particulates on the surfaces displayed in this study results from that of the aerosols that are inherited from the different sources of particles and their transportation in the Columbus module. The ISS’s aerosols surveys^38^ display a coarse particles fraction specifically formed under microgravity due to the absence of the sedimentation of particles >100 µm. Under microgravity, the transportation of aerosols is impacted by the absence of thermal turbulent flow (that is generated on Earth by changes in air density due to heat gradients), so in the laminar flows mixing is considerably reduced. Only Brownian motion, and electrostatic and phoretic^39^ interactions allow the motion of the particles and finally their contact with surfaces. Modeling particle deposits, both the fluid dynamics computation and the experimental^40^, thus requires an aerosol model that is not yet fully updated in low-level activity environments in particular in the Columbus Module^38,41^. The maximum acceptable values for the airflow are <450 m^3^ h^−1^ and for the concentration of particulate matter of the US Environmental Protection Agency of the National Ambient Air Quality Standards is 0.05–1 mg m^−3^ of particles <10 μm in aerodynamic diameter^42^. Considering these values, a maximum flow of 200 mg (down to 10 mg) over the glass lamella during the 6 months of exposure can be estimated. The measured values of particle density measured herein are several orders of magnitude lower than this corresponding calculated density, indicating particle concentrations maintained well below the tolerated threshold value in the Columbus Module and/or reduced fraction of the aerosols deposited on surfaces.

Seeing the diversity of the contaminating particulates on the surfaces displayed here also brings some insights to the potential types of foci and transmission routes of pathogens in the Columbus Module. A better understanding of them, coupled with that of the microbial loads of the different types of particles constitute a preliminary step towards modeling surface biocontamination. Surface microbial (bacterial and fungi) concentrations have been reported to fluctuate within a broad range, i.e., from 5 × 10^−3^ to 35 CFU/mm^2^ ^12^, though much lower or comparable to the concentrations reported in this study. However, these averaged values were evaluated by swabbing the surfaces. They therefore do not take into account the specific microbial loads of the different types of particles. This knowledge would be interesting to discover more efficient processes of surface cleaning.

Practically, manual sweeping is expected to be more efficient on the largest particles than on the smaller ones that interact more strongly with the surfaces. The observation of micrometer-sized particulates made in this study suggests that implementing hydrophobic coatings is an interesting approach to reducing surface biocontamination^32^. In terms of practical implementation of coatings for substantially larger areas of spacecraft building materials, other chemical treatments, such as surface polymerization deposited by different routes under atmospheric pressure can now be considered.

The necessity to cope with dormancy periods during unmanned phases, of one to two years for cis-lunar missions, and up to several years for Mars missions, is a critical aspect of near-future human spaceflights^43^. Aside from the need for passive contamination control hardware to contribute to the maintenance of an appropriate level of cleanliness of the spacecraft, fully automatized devices for microbial monitoring and control procedures while keeping the systems running at their minimum to decrease energy consumption will be required. A possible strategy to realize such sensors might consist of developing an advanced MATISS mechanical hardware to probe on the ground not only the number and size of the particles but also some information on their chemical and biological nature, using only non-invasive but penetrative radiations across the confined setup. This step would be of particular significance to the better address which technologies should be integrated into the designs of autonomous and miniaturized sensors to control contamination in situ in dormant spacecraft. A better knowledge of the nature of the ISS’s surface contaminations will be of benefit to their design.

## Methods

### Surface coatings

For this study, we selected nano particle-free surface coatings deposited with solvent-free automatized techniques, compatible with a wide range of materials, including glass slides, and with hydrophobic surface properties. The selected chemical vapor deposition processes were all carried out in a vacuum, which has the advantage in a proof of concept carried out on glass lamella to limit the use of potentially toxic organic solvents and to provide an excellent intra and inter lot reproducibility.

FDTS coating is based on (1H,1H,2H,2H)-perfluorodecyltrichlorosilane FDTS (ABCR, 97%). The coating application was performed using commercially available molecular vapor deposition equipment (MVD100 from Applied MST, San José, US). The deposition conditions for FDTS were as follows. In a first step, the surface was cleaned using remote RF oxygen plasma (450sccm O_2_ flow, 250 W, 300 s). In a second step, one cycle of tetrachlorosilane SiCl_4_ (Sigma Aldrich, 99,998% Semiconductor grade) at 18 Torr was injected, followed by four cycles of water at 18 Torr. This step took place for a duration of 600 s at 35 °C. In a third step, two cycles of FDTS at 0.5 Torr were injected, followed by one cycle of water at 18 Torr. This step took place for a duration of 900 s at 35 °C and aimed at grafting FDTS to the surface by a silanisation reaction. The optical thickness of the FDTS layer extrapolated from measurement obtained by Surface-Enhanced Ellipsometric Constrast technique on thermally oxidized silicon substrates was 1.6 ± 0.2 nm. The water contact angle, measured by goniometer equipment from GBX instruments, on these layers of FDTS was ~110 ± 2°.

SiOCH thin films were deposited onto a 200 mm radiofrequency capacitive-coupled parallel-plate reactor from Applied Material (using a plasma excitation frequency at 13.56 MHz). Octamethylcyclotetrasiloxane (OMCTS), obtained from Sigma-Aldrich, was used as precursor, as-received without any further purification. Depositions were performed under vacuum (2 Torr) at 100 °C and film thicknesses after deposition were firstly measured by spectroscopic ellipsometry on silicon wafers before deposition onto glass substrates. The thickness of SiOCH thin films was 1 µm. The water contact angle, measured by goniometer equipment from GBX instruments, on these layers of SiOCH, was ~105 ± 2°.

The Parylene layer was deposited using Vapor Deposition System PDS 2010 Labcoter® 2 from SCS with Parylene C (Dichloro-di-para-xylylene) as a precursor. The deposition conditions for the Parylene layer were as follows. First, dichloro-di-para-xylylene was sublimated at 150 °C under vacuum (1 Torr). Then, pyrolysis occurred at 680 °C under vacuum (0.5 Torr). Finally, the deposition was performed at 25 °C under vacuum (0.1 Torr). The thickness of the Parylene layer, measured by spectroscopic ellipsometry, was 5 µm. The water contact angle, measured by goniometer equipment from GBX instruments, on these layers of Parylene was ~87 ± 4°.

### Sampling with the MATISS sample holder

MATISS sample holders were mounted with Parylene, FDTS and SiOCH coated surfaces, sealed with Kapton tape, and placed into two Ziploc bags using gloves. They were brought into the Columbus module by Cygnus CRS OA-5 on 17 October 2016 as part of the MATISS experiment. They were mounted by an astronaut in two sites with a low frequency of astronaut contact and good airflow. Two holders were mounted in the direct vicinity of the Return Grid Sensor Housing (Supplementary Fig. 5A), the most important intake of air in the Columbus Cabin. This Grid is located near the hatch and sucks in air at a rate of 400 m^3^/h. Part of this air is re-injected into the cabin, while the majority is sent to the next module. Air velocity was modeled at 0.21 m/s (∼40 ft/min = 0.21 m/s). Crew activity in this location is carried out quickly and limited to maintenance or cleaning tasks. One holder was mounted on the surface of the EPM Rack front panel (Supplementary Fig. 5B). This rack is located in the middle section of the Columbus cabin. The main source of airflow in this location is an air out-take located ~1 m above the exposure location blowing 55 m^3^/h of air at a speed of 0.21 m/s. The flow is not directed towards the rack surface but towards the center of the cabin. Crew activity around this location is regular. They were exposed to air by removing the Kapton tape on 21 November 2016. Sample holders were sealed with Kapton tape using gloves, placed into two Ziploc bags and stored at room temperature the day before the return with the Soyuz 49S on 2 June 2017. Samples were transferred on 9 June 2017 to our laboratory, avoiding X-ray scans and using temperature monitoring to ensure that no inadvertent extreme temperature events were applied to the sample holders (values in the range of 5–50 °C). They were stored at 5 °C.

### Optical microscopy and image analysis

The MATISS sample holder was mounted on a raster X-Y table and a tile scanning mode was applied to image the full glass surface visible across the polycarbonate cover using the optical macroscope MacroFluo Leica Z16 ApoA and a PlanApo 5 × /0.5 coupled to a QImaging QICAM fast 1394 camera (12 bits, 1392 × 1040) controlled by a MetaMorph interface. A stack of 30 RGB images (75 ms exposition time) was produced at low zoom (×3), and of 1452 images (100 ms exposition time) at high zoom (×30).

We developed the processing of the stack of images that provided identifications and optical measurements for each particle with additional data, such as position, area, and elongation ratio. The output was a table listing each particle found and the features of those particles. For the stack recorded at low zoom (Supplementary Fig. 6), the segmentation of the image was performed on the blue component using a constant threshold value of gray level of about 75 that was empirically determined. For the stack recorded at high zoom (Supplementary Fig. 7), the processing of the images containing macroscopic objects with shadows masking the small particles, were removed. The mean intensity of the blue image was compared to the median value (background) incremented by two times the average value of the standard deviation of the intensity of the stack. The positions of every particle were determined using the Analyze Particle module of FIJI. Based on these positions, crops were recorded for each particle and the segmentation was refined using a local threshold value that was a function of the mean value of the crop. At high zoom, crops were collected on an 8-bit sum of the RGB images, while high zoom crops were sampled on the blue image. The area and the elongation of every particle were then determined using the Analyze Particle module of FIJI.

### Reporting summary

Further information on research design is available in the Nature Research Reporting Summary linked to this article.

## Supplementary information

Supplementary Figures

Reporting Summary Checklist FLAT

## Data Availability

The dataset of particle areas analyzed during the current study are available from the corresponding authors on reasonable request. The MATISS sample holder was deposited at the Institut National de la Propriété.

## Code availability

The 2 codes generated during the current study, that are described in the Supplementary Figs. 6 and 7 are available from the corresponding authors on reasonable request.
