# Supplementary material for: Towards a passive limitation of particle surface contamination in the Columbus module (ISS) during the MATISS experiment of the Proxima Mission
Source: NPJ Microgravity. 2020 Oct 20;6:29. doi: 10.1038/s41526-020-00120-w (PMC7576818; doi:10.1038/s41526-020-00120-w)
Supplement: Supplementary file 1 — Supplementary Figures [file 41526_2020_120_MOESM1_ESM.pdf]

Supplementary Figures

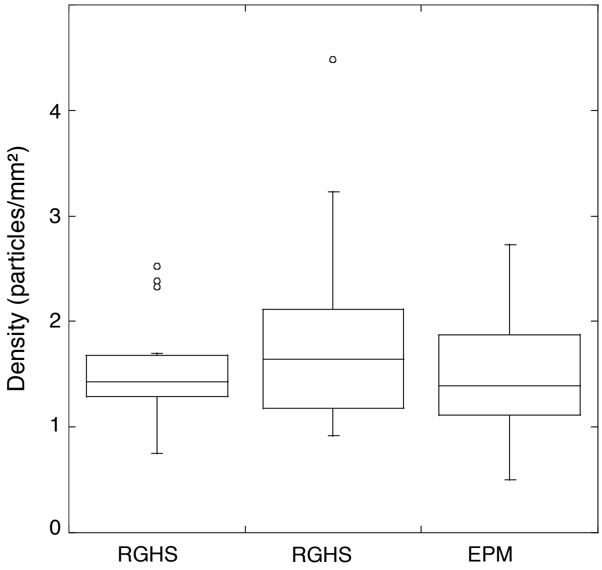

Supplementary Figure 1: Surface contamination in different ISS locations. Box-plots showing the density values of the coarse particles (50 < Area < 1500 mm^2) measured on 16 area of 60 mm^2 for each location. Centerlines show median, and edges the first and third quartiles. “Whiskers” extend to the largest and smallest data points within 1.5 interquartile ranges of the first and third quartile. Two sample holders were exposed near the Returning Grid Sensor Housing (RGHS) and one sample holder on the EPM rack front panel. Statistical differences between the three distributions were examined by unpaired Student’s t-test and were not found to be significantly different ($p > 0.18$)

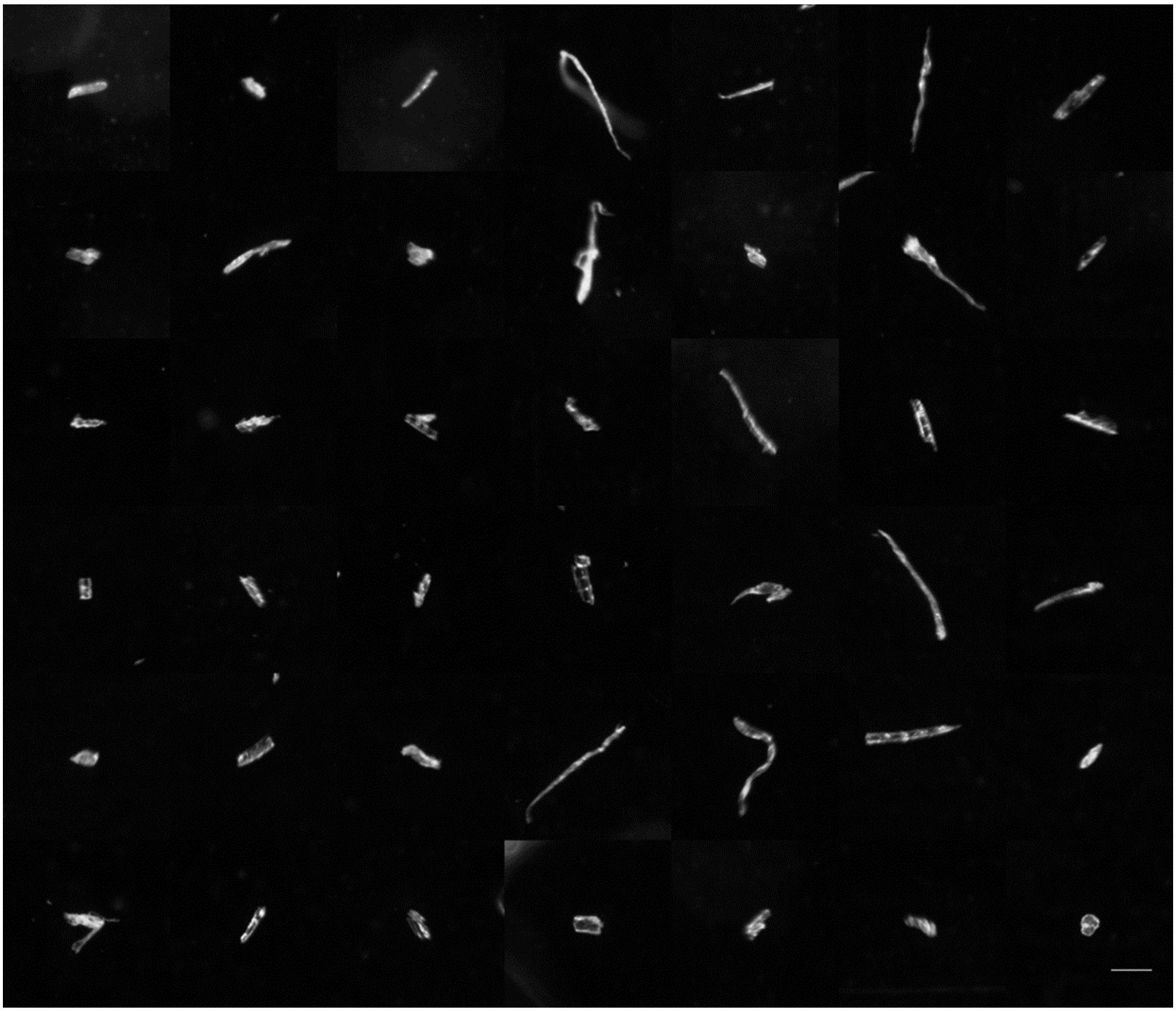

Supplementary Figure 2: Surface contamination by coarse fiber particles. Mosaic of optical images displaying typical shapes of fibers. Scale bar is 100 µm.

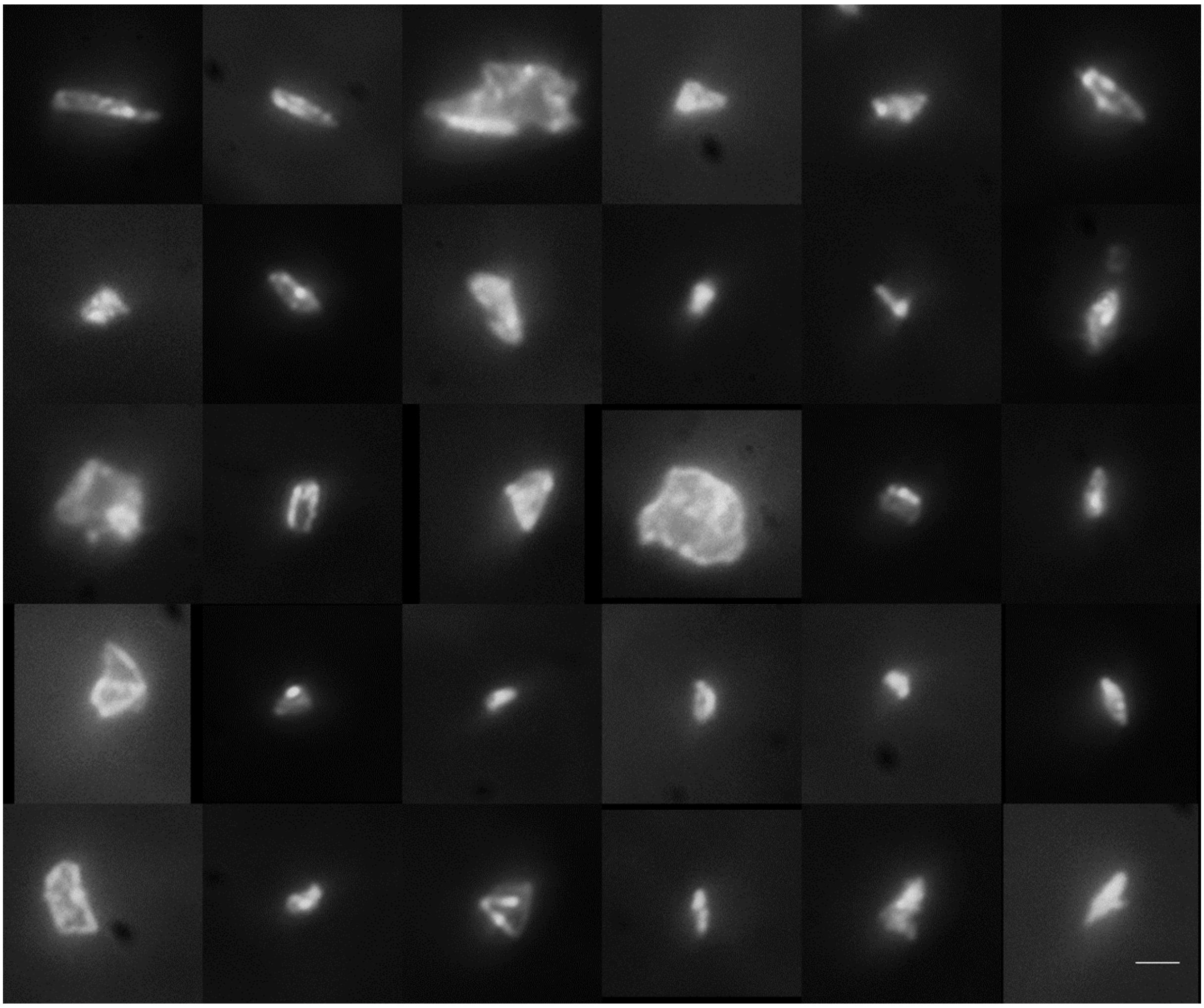

Supplementary Figure 3: Surface contamination by coarse particles. Mosaic of optical images displaying single particles with an area higher than 50µm² and lower than 1500µm². Scale bar is 10µm.

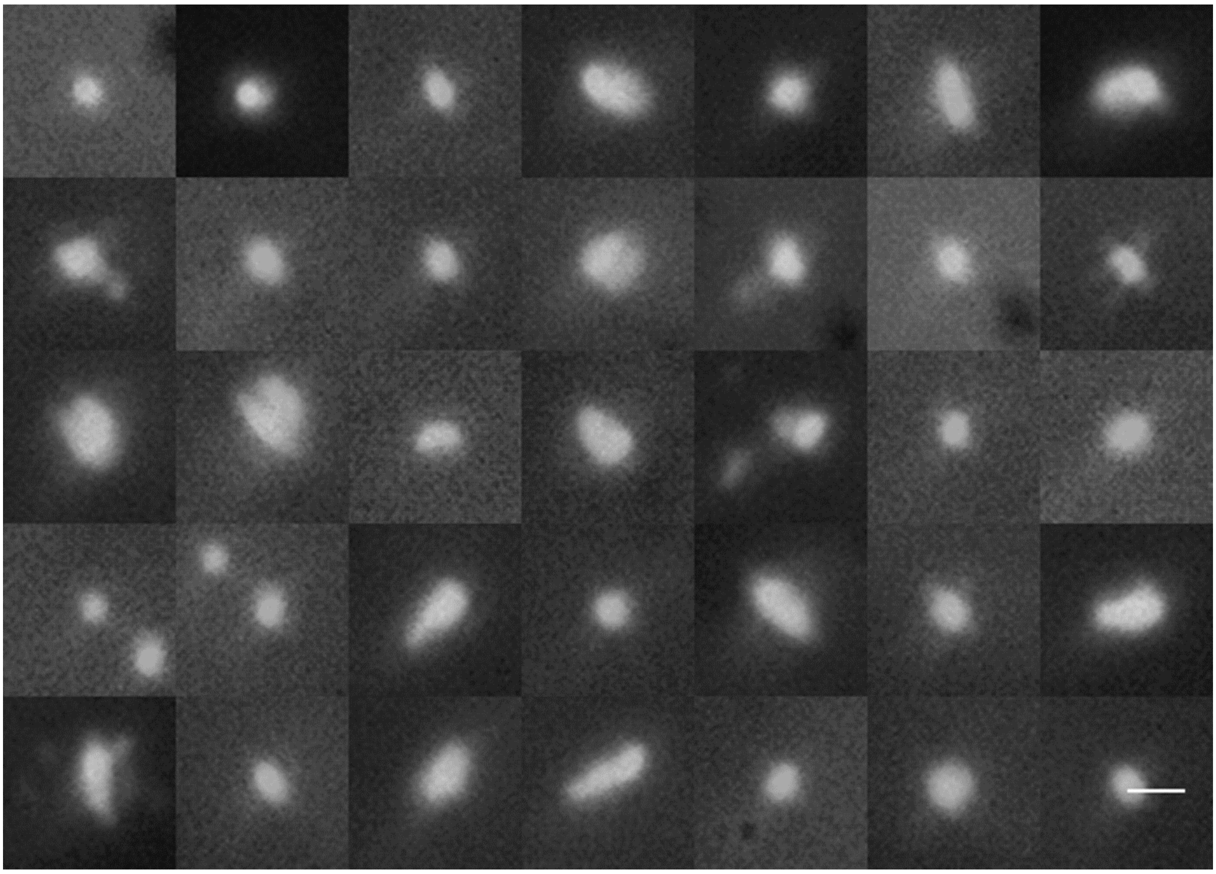

Supplementary Figure 4: Surface contamination by fine particles. Mosaic of optical images displaying single particles. Scale bar is 5μm.

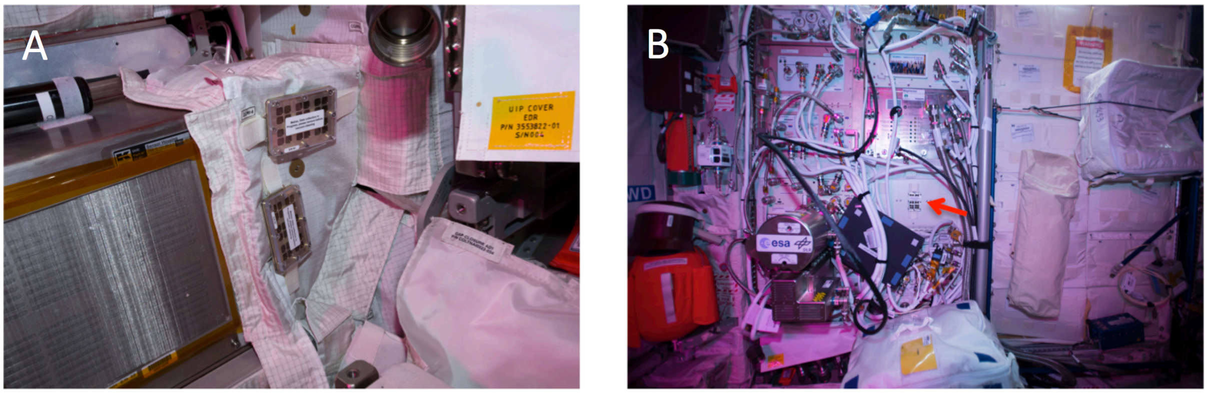

Supplementary Figure 5: Photographs of the sample holders once installed in the Columbus module. (A) The two holders installed near the Return Grid Sensor Housing (RGHS) and (B) the holder installed near the European Physiology Modules Facility (EPM) front panel. Holders' size **:** 8.5cm × 6cm × 1.2 cm. Photograph courtesy of NASA/ESA permissible to use within the public domain.

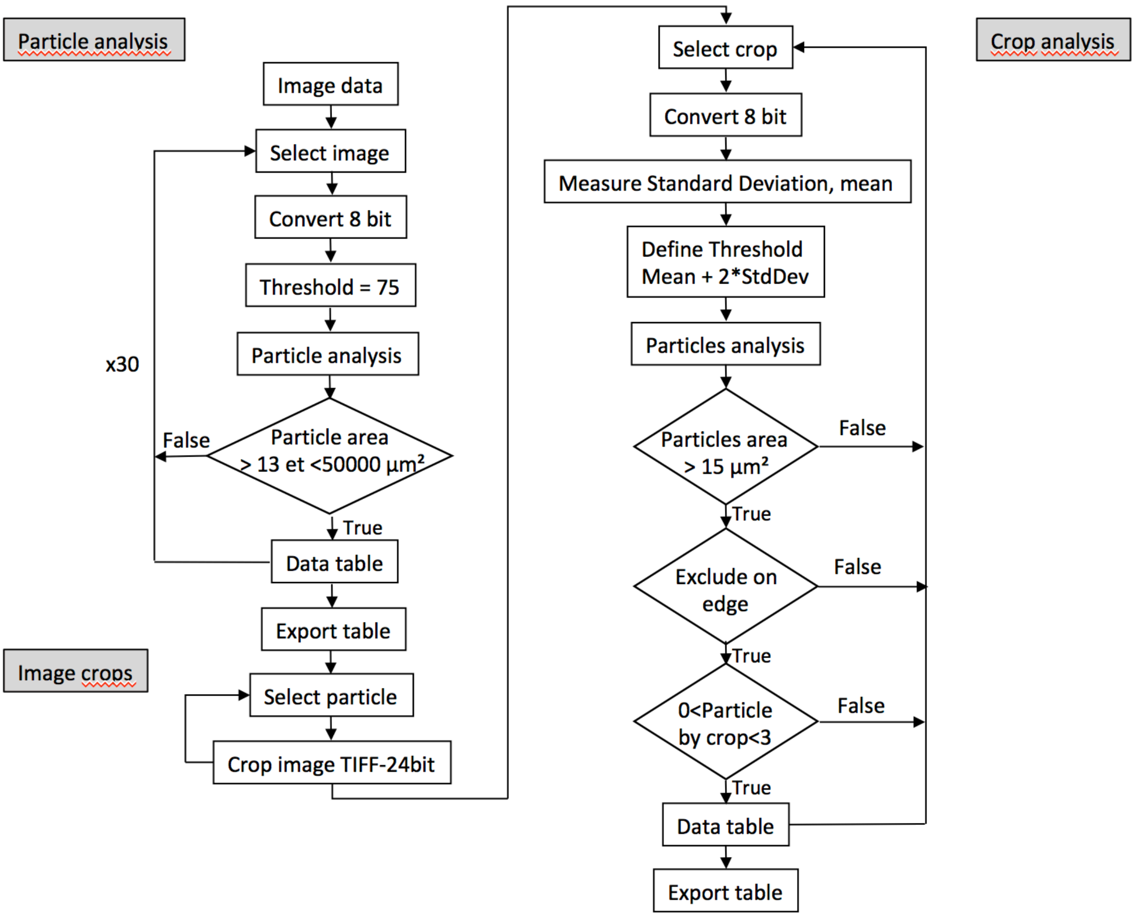

Supplementary Figure 6: Flowcharts of the processing of the stacks of images recorded at low zoom.

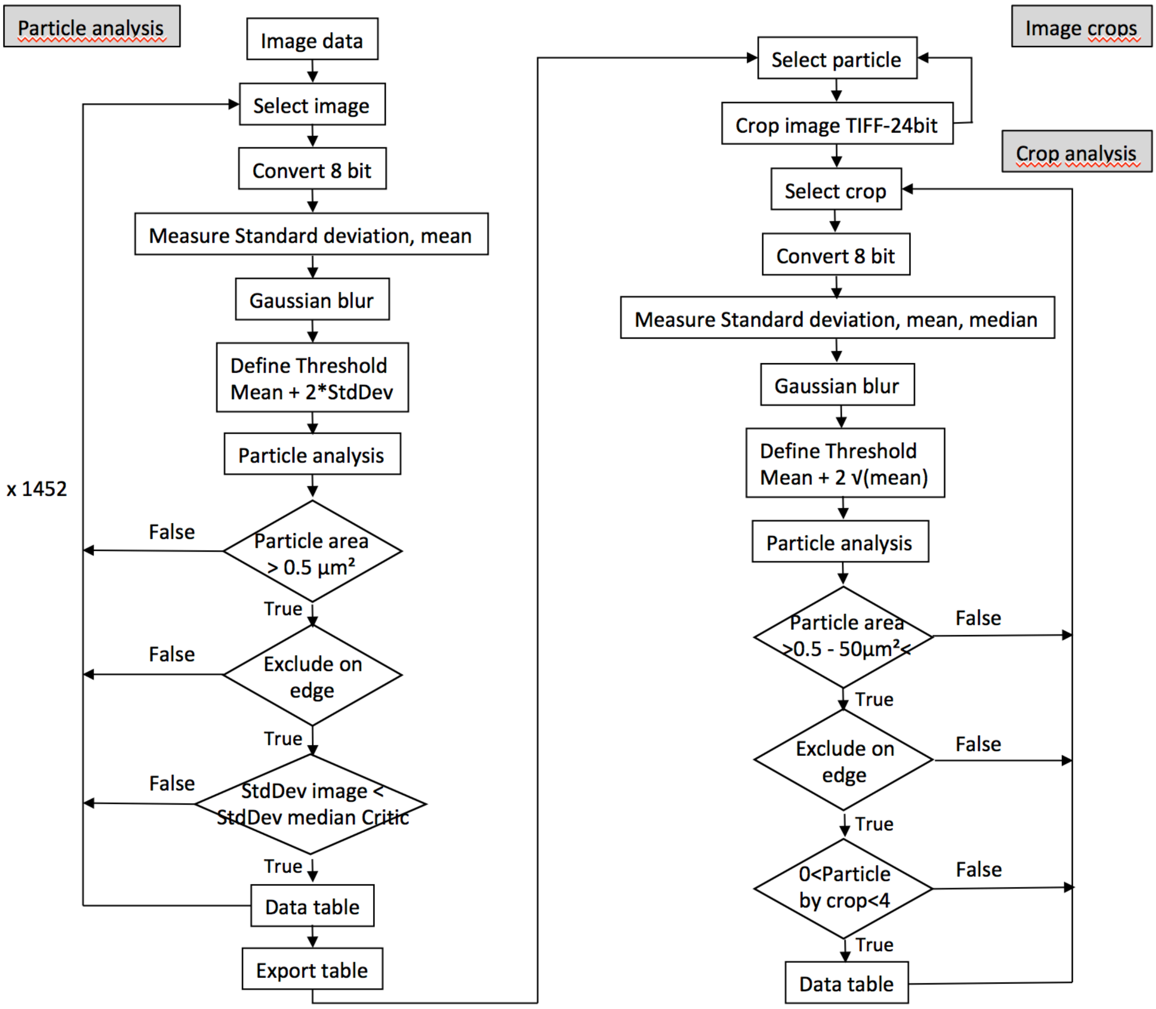

Supplementary Figure 7: Flowcharts of the processing of the stacks of images recorded at high zoom.
